# Torture and Maltreatment in Prison: A Medico-Legal Perspective

**DOI:** 10.3390/healthcare11040576

**Published:** 2023-02-15

**Authors:** Giuseppe Davide Albano, Daniela Guadagnino, Mauro Midiri, Corinne La Spina, Valeria Tullio, Antonina Argo, Stefania Zerbo

**Affiliations:** 1 Section of Legal Medicine, Department of Health Promotion, Mother and Child Care, Internal Medicine and Medical Specialties, University of Palermo, 90129 Palermo, Italy; 2 Interdepartmental Center of Research (CIR) “Migrare” on Migration, University of Palermo, 90129 Palermo, Italy

**Keywords:** torture, maltreatment, ill-treatment, prison, custody, jail, medico-legal issues, forensic, autopsy

## Abstract

The maltreatment and torture of prisoners constitute a global problem. Methods of maltreatment are classified as the psychological and the physical, and physical methods inevitably lead to psychological sequelae. Our review offers an analysis from the medico-legal perspective of the literature on the torture and physical and sexual abuse experienced by prisoners and their psychological sequelae and aims to investigate the medico-legal issues of investigating maltreatment in prison so as to suggest methodologies and updated approaches for dealing with such cases in a forensic context. We performed a comprehensive literature search of peer-reviewed publications (articles and reviews), research reports, case studies, books, service models, protocols, and institutional documents available online using key electronic databases (Scopus, PubMed) and search engines (Google Scholar) with the following keywords: physical violence, psychological violence, torture, maltreatment, physical abuse, psychological abuse AND prison OR prisoner OR jail OR custody. In the medical literature, most of the publications on torture are based on retrospective studies of torture among survivors and often refer to asylum seekers. Forensic evaluation is crucial for assessing the determinant elements of torture and maltreatment. A multidisciplinary approach and standardized and updated methodologies are needed to support policymakers, national institutions, and public health system initiatives in this field.

## 1. Introduction

The abuse and torture of prisoners constitute a global problem. In prisons, because of the subjugation-induced lack of liberty, detainees are particularly at risk of being victims of torture or ill treatment [1]. 

Art.1 of the Declaration on the Protection of All Persons from Being Subjected to Torture and Other Cruel, Inhuman or Degrading Treatment or Punishment, provided by the General Assembly resolution in 1975, defines torture as “any act by which severe pain or suffering, whether physical or mental, is intentionally inflicted by or at the instigation of a public official on a person for such purposes as obtaining from him or a third person information or confession, punishing him for an act he has committed or is suspected of having committed, or intimidating him or other persons. It does not include pain or suffering arising only from, inherent in or incidental to, lawful sanctions to the extent consistent with the Standard Minimum Rules for the Treatment of Prisoners” [2]. 

Methods of maltreatment are classified as psychological and physical, of which physical methods inevitably lead to psychological consequences. Some maltreatment methods, such as sexual violence, almost unavoidably combine physical abuse with psychological abuse [3,4,5,6]. 

Violence is an integral part of prison life [7,8,9]. In extreme cases, violence can result in homicide [10]. In 2000, inmate physical attacks in federal or state prisons led to 51 deaths (less than 0.1 per 1000), down from 82 in 1995. In prisons, homicides are rare compared to inmate-on-inmate physical assault (i.e., slapping, hitting, kicking, biting, choking, or beating) [10]. 

Detainees may be victims of violence inflicted by prison staff or fellow inmates. According to official statistics, in 2000, for every 1000 inmates in prisons, a prisoner committed 28 physical assaults [11]. Prisoner-on-prisoner violence must be distinguished from staff-on-prisoner violence and torture in prison. Inmate–inmate altercations are usually related to structural and interpersonal variables. Inmate–staff altercations are often related to the extent to which inmates are involved in social relationships with other inmates and percievethe correctional staff as a physical threat to them. Torture is a subgroup of collective violence, defined specifically by the severity of the pain, the intentionality, the purpose, and the perpetrator. Torture is prohibited by international law, and there are no circumstances that justify an exception to this prohibition. Nevertheless, according to human rights reports, torture is practised in about 130 countries and is widespread and systematically used in 80–100 countries [4,5,6,7,8,9]. The authorities must, therefore, ensure the protection and humane treatment of prisoners throughout their detention. Some more vulnerable detainees, particularly those affected by mental disease, have special needs (and they maybe more exposed to the risk of torture). For this reason, they require additional protection from international, national, and local authorities to ensure that their human rights are respected [1]. In addition, as a form of deprivation of personal freedom, detention itself can be considered as ill treatment or even torture under certain circumstances, in terms of human rights [12]. 

Even though physical violence is assumed to be prevalent in prisons [9], the related data are limited. Not much is known about the epidemiology and context of physical violence inside prisons, and even less is known about the link between mental disorders and physical abuse [10]. Maltreatment in custody is still a debated topic worldwide and a policy and public health issue for which forensic sciences play a relevant role in providing proof of quality to the decisionmakers. In this regard, forensic investigation and its methodology (information sources, cross-checking, forensic examination of the body, inspection of facilities, medical history analysis with healthcare workers, and analysis of medical records) play key roles in the fact-finding process and in allegations of maltreatment in custodial settings. The allegation of maltreatment depends on several factors: the socio-demographic context, national legislation, international conventions, control by local activists and international organizations, and the reporting of international and national media.

Our review offers an analysis from the medico-legal perspective of the literature on the torture and physical and sexual abuse experienced by prisoners and the psychological consequences with a focus on staff-on-inmate violence, the goal being to pay attention to the protection and respect of human rights and to explore the still lacking literature in this field. Furthermore, this review aims to investigate the medico-legal issues of investigating maltreatment in prison so as to suggest methodologies and updated approaches for dealing with such cases in the forensic context.

## 2. Materials and Methods

We performed a comprehensive literature search of peer-reviewed publications (articles and reviews), research reports, case studies, books, service models, available protocols, and institutional documents available online using key electronic databases (Scopus, PubMed) and search engines (Google Scholar) with the following keywords: physical violence, psychological violence, torture, maltreatment, physical abuse, psychological abuse AND prison OR prisoner OR jail OR custody. No temporal limit was established for the literature search. The research was completed in October 2022. We screened the articles by title and abstract in full text, if relevant. The search was then expanded to reference lists from the published key articles, if relevant. Subsequently, a full-text evaluation of the selected studies was carried out. On the basis of the literature search, we identified 2006 studies. The quality of each study was evaluated independently by three authors (G.D.A., D.G., and M.M.). If there were conflicting opinions regarding any article, it was submitted to A.A. We defined prison settings as prisons, jails, and other custodial settings. The prison populations were all incarcerated adult populations (≥18 years). We included only articles in English with available full-text documents. The only exclusion criteria were unspecific articles and those not written in English language. Finally, we included 50 articles in this review.

## 3. Results

### 3.1. Physical Abuse

The lifetime prevalence rates of physical and sexual violence are higher among inmates, at 6 months and 12 months of incarceration, than among non-prisoners. In addition, women report much higher rates of traumatic events than men [13]. 

The physical consequences of maltreatment depend on the duration and periodicity of the methods, the amount of force used, whether the victim can protect himself or herself, and the prisoner’s prior health status [3,4,5,6,12]. 

Several studies have focused on the physical effects of maltreatment in custody, showing that maltreatment can have immediate, short-term, and long-term functional effects and can be strongly associated with known maltreatment methods [3,4,5,6]. However, some maltreatment methods, such as plastic bag asphyxiation and near-drowning, do not result in physical injuries [3,4,5,6]. 

Because the methods of mistreatment are highly diverse with considerable geographical variability, no strict forensic classification has emerged. The procedures and frequency of such phenomena are related to the social and cultural contexts, and these factors need to be taken into consideration in a forensic evaluation. In this regard, Edston analyzed the physical signs of torture in Swedish asylum seekers, suggesting that some forms of physical violence in prison, such as falanga (beating on the soles of the feet), are relatively specific to particular geographic areas, such as the Middle East [14,15]. Some methods are specific to countries: Pounder et al. focused on shaken adult syndrome, highlighting that such a manner of torture is frequent in Israel [16], while Morentin et al. evaluated the torture methods used in in Basque countries by the Spanish anti-terrorist police, suggesting an increased use of plastic bag asphyxiation [17]. According to Vogel et al., torture in custody can be differentiated intonon-life-threatening maltreatment (with or without mutilation), life-threatening abuse, and maltreatment meant to kill [18]. Crush injuries were only reported by refugees from Asia, including Afghanistan and Pakistan, and incidents of electrical torture were reported twice as frequently by torture victims from Middle Eastern and North African countries, though these incidents were observed at a lower rate among Iraqis, Iranians, and ethnic Kurds. Sexual torture was reported by 78% of females and 25% of males. The most common methods worldwide are beatings, electric injuries, several forms of asphyxia, suspension, thermal injuries, and sexual violence [3,4,5,6,14]. The following sections summarize the characteristics of the most frequent types of maltreatment and torture in custodial settings.

#### 3.1.1. Blunt Force Trauma

**Figure 1:**
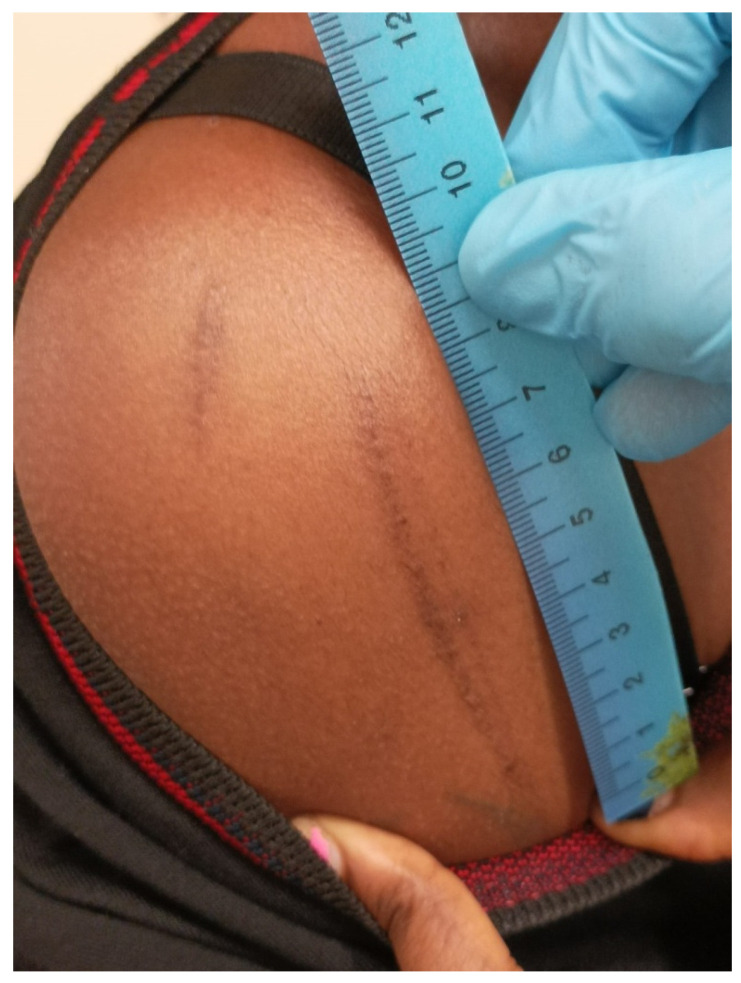
Bruise scars inflicted with a whip on the shoulder of a young female in prison (forensic inspection performed at the Medico-Legal Unit of the University of Palermo).

**Figure 2:**
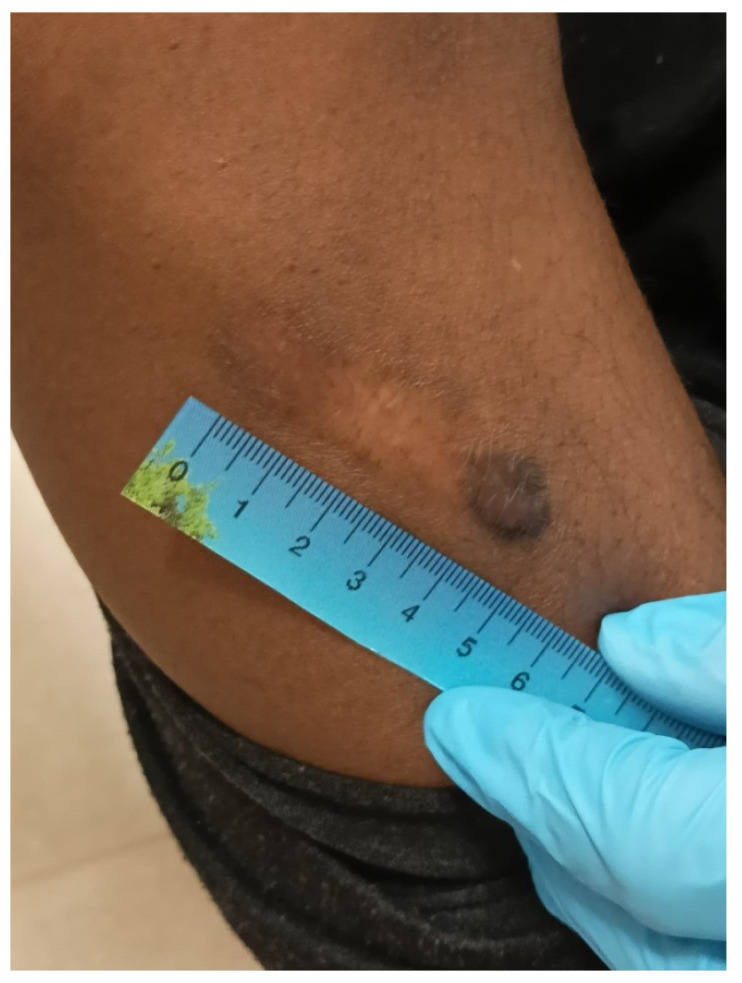
Blunt force scar inflicted on the right arm (plastic tube) of the same patient in prison (forensic inspection performed at the Medico-Legal Unit of the University of Palermo).

**Figure 3:**
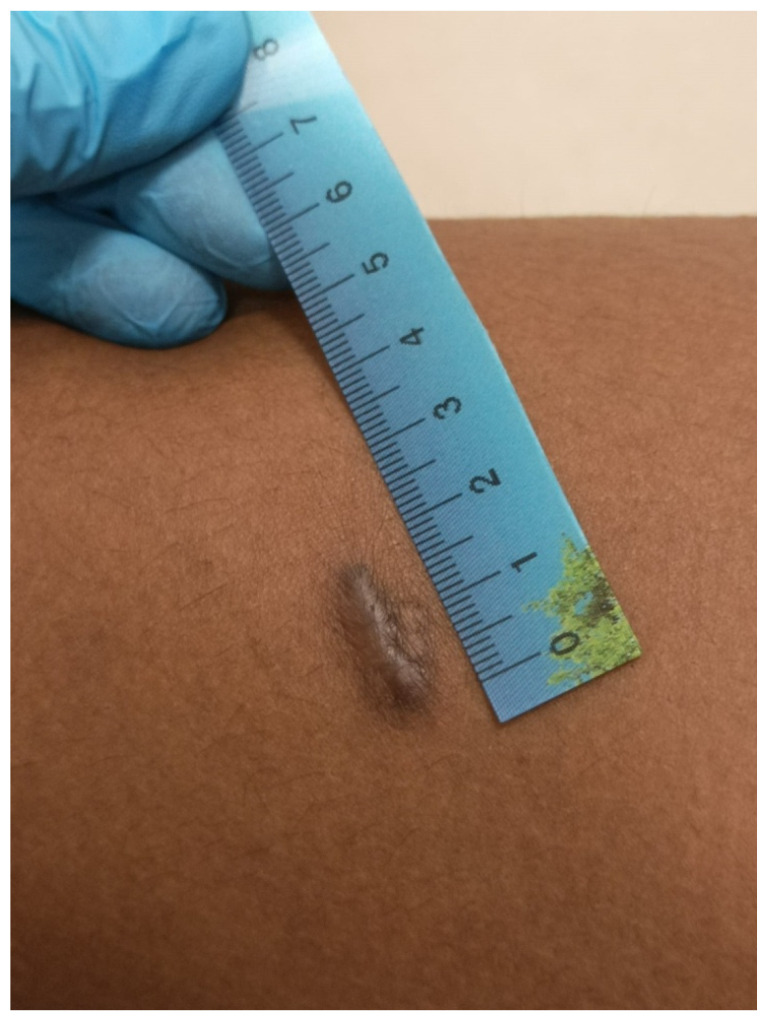
Blunt force scar inflicted on the leg (plastic tube) of the same patient in prison (forensic inspection performed at the Medico-Legal Unit of the University of Palermo).

The use of blunt force is a frequent manner of abuse. Physical injuries are often nonspecific, but blows from batons, whips, electric cables, and sticks commonly leave classic “track” bruises or permanent hypo- or hyperpigmented scars (Figure 1, Figure 2 and Figure 3) [3,4,5,6,19]. 

Beatings are a common occurrence applying to all areas of the body [18]. The most common form of torture is hitting on the head, which can induce intracerebral and facial sinus bleeding and fracture the facial bones. The intensity and aim of such beatings determine the risk. Therefore, death by accident can occur [18,20]. Following chronic exposure to head concussion, a torture survivor may complain of continuous headaches. Depending on the force and method, blows to the trunk may cause rib fractures, which, if decomposed, may be associated with lung lacerations and possible pneumothorax. Acute abdominal trauma may cause edema, contusion, hemorrhage, or organ laceration. Falanga is the most common term for repeated blunt trauma to the feet (or, more rarely, the hands or hips), usually applied with a baton, a pipe, or a similar weapon. It may result in chronic disability, making it painful and difficult to walk. According to the United Nation’s Manual on the Effective Investigation and Documentation of Torture and Other Cruel, Inhuman or Degrading Treatment or Punishment, the use of falanga is typical in the Near East, especially in Turkey, Iraq, the Far East, and some Spanish-speaking areas [12,18]. The most serious complication is closed compartment syndrome, which can cause muscle necrosis, vascular obstruction, bone injuries or gangrene affecting the distal part of the foot or toes. Other complications are the crushing of the heel and anterior plantar pads and stiffness [21]. 

#### 3.1.2. Electric Injuries

The application of electricity for abuse is also a common practice worldwide. Ozkalipci et al. studied the most frequent findings of medical assessments of torture, focusing on electric injuries, in depth [22]. The medical history of the victim should provide information about the location and the source of electricity so that the site maybe visited and the equipment searched. If prisoners are hooded or blinded, they may only heard the sound of the device. The immediate description of the primary consequences, such as tetanic contraction and pain, is crucial, as is information about the position of the electrodes and the type of electrodes, such as alligator clips, cut ends of electrical wires, or wires wrapped around fingers. The most common places to which electrodes are applied are the area between the toes and the tongue, teeth, penis, anus, and vagina. When electrodes are applied between the toes or to the tongue, the place of entry of the electric current is hidden, while application to the penis inflicts pain and humiliation [3,4,5,6,18,22]. 

In a recent study that focused on allegations of maltreatment in custody, Vogel et al. showed that in Africa, electrodes are positioned on the teeth. Moreover, they observed that in the Middle East, perpetrators place large electrodes on wet skin and collars placed around the neck [18]. 

In acute cases, an electric current can cause tetanic contractions and muscle damage, with the onset of myoglobinuria. A moderately high current leads to shoulder dislocation and lumbar and cervical radiculopathy [12]. 

It is important to evaluate any electrical signs that may be present on the skin and describe their initial appearance. Electric marks are reddish-brown in color with inflamed margins, before they darken, resolve completely, or leave thin white scars, which are punctiform in cases when a wire end has been used [3,4,5,6,12]. These might be partially circumferential, wrapping around a finger, if the wire was wrapped around it. Clusters of punctiform scars (picana) on unusual locations, including the toes or the foreskin, are strongly suggestive and typical of maltreatment using electric current. Still, these scars are small and, therefore, may generally remain undetected unless explicitly pointed out by the detainee [3,4,5,6,18]. 

#### 3.1.3. Asphyxiation

Suffocation is used in many forms. It usually leaves no marks, and recovery is rapid. As suggested by Saukko et al., only in rare cases can it result in fractures of the larynx or soft tissue scarring, which can aid in medico-legal evaluation [19]. Near-asphyxiation by suffocation is a widely used method of torture in Latin America. Its name in Spanish, submarine, has become part of the vocabulary of human rights. Normal breathing can be prevented by covering the victim’s head with a plastic bag, closing the mouth and nose, pressing the neck or tying something around it, or forcing the victim to suck in corpuscular material (such as dust). This phenomenon is also known as “dry submarining”. Another method used may be forced immersion of the head in water, which can result in near-drowning or drowning. This form of torture is known as “wet submarining”. In hanging or other forms of ligature asphyxiation, abrasions or bruises are often found on the neck. The hyoid bone and laryngeal cartilage may be fractured due to partial strangulation or blows inflicted on the neck [3,4,5,6,12]. However, such findings are relatively infrequent.

#### 3.1.4. Forced Positions and Suspension

**Figure 4:**
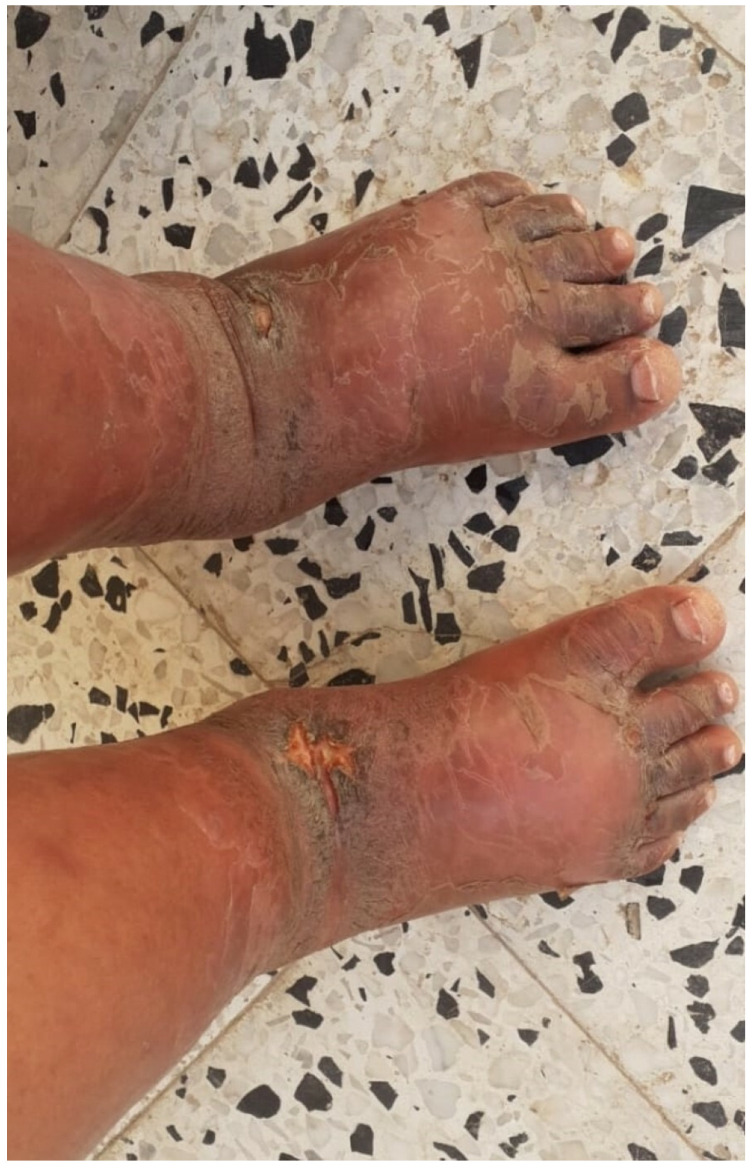
A young female who arrived in Sicily through the humanitarian corridor of the Italian government (forensic inspection performed at the Medico-Legal Unit of the University of Palermo). Symmetrical scars from instep bindings inflicted in prison. Concomitant severe lymphedema treated in a Libyan hospital after release.

Forced positions are applied worldwide in prisons, as reflected in the literature. As reported by Woldu et al., 15–63% of all torture survivors report having been suspended [16]. These instances can last for hours or even days [18]. There are many forms of positional torture that bind or restrain the victim in twisted, hypertensive, or unnatural positions, which can cause tendon, joint, and muscle injuries. The various methods include parrot suspension; the banana stand, i.e., the classic banana tie on a chair, whether on the floor or on a motorcycle; a forced standing position; forced one-footed position; prolonged standing position with the arms and hands stretched high against a wall; and prolonged forced squatting and forced immobilization in a small cage. Among these forms, suspension is the most common. The original method for suspension is the strappado, which derives from the Inquisition [3,4,5,6,16,23]. In this method, the wrists of the victim are tied behind the back, and then the victim is suspended by the wrists. Today, this method is sometimes referred to as “Palestinian hanging”, associating a method of torture with a specific geographic area. The strappado method leads to severe pain and is related to the loss of consciousness in a short time, and the immediate residual effect is frozen shoulders. This resolves with pain, muscle weakness, and numbness, or it may resolve completely. When the suspension is asymmetrical, it can have asymmetrical effects on the two arms. Brachial plexus injury is the most severe, with permanent motor and sensory damage. Moreover, the ligature and the suspension can lead to lymphatic drain impairment and, therefore, lymphedema (Figure 4) [3,4,5,6,12]. In prison, residual motor or sensory deficits from the suspension can result in difficulties in eating, dressing, diuresis, and defecation for the inmate, leading to requests for assistance from fellow inmates and associated humiliation.

All forms of suspension or ligature involving limb ligation can cause scars and ligature marks of specific types in specific locations (Figure 4). Suspension is almost always accompanied by other forms of torture, such as suffocation, electric injuries, and blunt force trauma [12,22,23]. 

#### 3.1.5. Thermal Injuries

**Figure 5:**
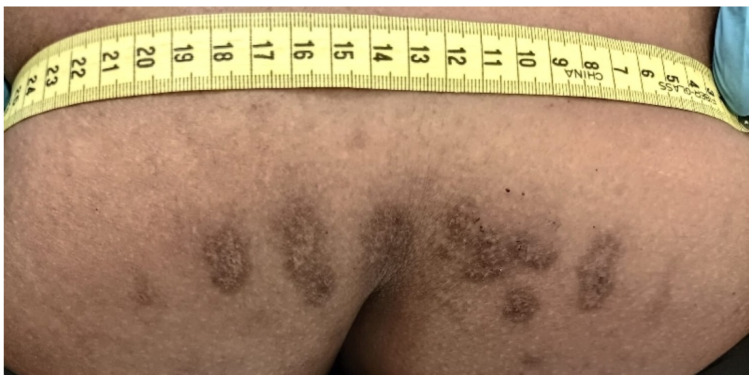
Multiple bilateral macular oval-shaped scars as a consequence of cigarette burns on gluteal skin (forensic inspection performed at the Medico-Legal Unit of the University of Palermo).

Burning is the most frequent form of torture that can leave permanent changes on the skin, which can sometimes have diagnostic value [19,22]. Cigarette burns often leave 5 to 10 mm-long circular or oval macular scars. They may have a hyper- or hypopigmented center and a hyperpigmented, relatively indistinct periphery. Cigarette tattoo burns and burns caused by hot objects related to torture have also been reported. The specific shape of the resulting scars and any tattoo remnants aid in diagnosis. In particular, burning with hot objects produces markedly atrophic scars that reflect the tool’s shape and are sharply demarcated by hypertrophic or hyperpigmented marginal areas that are related to the initial zone of inflammation (Figure 5). This may result, for example, from a burn inflicted using an electrically heated metal rod or a gas lighter. However, if many scars are present, it is often difficult to perform a differential diagnosis. In addition, the burn may result in hypertrophic or keloid scars, as in the case of a burn caused by burning rubber [12]. Traumatic victimization, particularly sexual abuse, has consistently been identified as a problem within jails [3,4,5,6]. Table 1 summarizes the most frequent methods of maltreatment and torture in custody.

### 3.2. Sexual Abuse

Sexual abuse has been consistently identified as a high-frequency problem in prisons [13]. Sexual abuse can be broadly defined as including violence against sexual organs, including the introduction of foreign bodies into the vagina or the rectum, rape, and other forced sexual acts, and mental sexual violence, such as forced nudity, sexual humiliation, sexual threats, and forced witnessing of sexual abuse. The prevalence of sexual abuse is high among victims of prison maltreatment [24,25,26,27,28,29,30,31]. Neal et al. showed that various factors make it difficult to carry out fact-finding missions. Sexual torture can begin with forced nudity, a constant factor of torture situations in many countries. Verbal sexual threats, abuse, and teasing are also part of sexual abuse. All of these elements contribute to humiliation and degradation [25]. 

There are some differences between the forms of sexual torture applied to men and women. Rape in men’s prisons was first recognized as a crime by the U.S. Supreme Court in Farmer v. Brennan (1994) [24], which unanimously ruled that the Eighth Amendment’s prohibition of cruel and unusual punishment has been violated in cases where prison guards acted with “deliberate indifference” and “ignored a substantial risk of serious harm” to the inmate, noting that sexual abuse “is not part of the punishment that criminals pay for their crimes against society”.

Sexual victimization has become so widespread and alarming that in 2003, the U.S. Congress passed the Prison Rape Elimination Act (PREA) to identify, prevent, prosecute, and respond to sexual violence in correctional institutions [25]. According to Barom, the phenomenon of sexual assault victimization among female prisoners is still not adequately debated and explained.

Sexual violence in prison can have serious and lasting implications, with potentially devastating physiological, social, and psychological consequences [26]. Rapes can be violent, bloody, and physically traumatic for victims. Even worse, gang rapes are often characterized by extreme violence and can be even more traumatic, as reported by a human rights report of 2006 [27]. 

Rape can be associated with the risk of developing sexually transmitted diseases, particularly human immunodeficiency virus (HIV), and unwanted pregnancies for women [28,29,30,31]. HIV infection rates are higher in prisons than in the general population. Lunde at al. stated that the only effective HIV prophylaxis must be taken within hours of the event, and unfortunately, it is not generally available in low-income countries where torture routinely occurs [12,32]. 

Several studies have demonstrated that sexual victimization can lead to future violence inside or outside prison; depression; and acts of hetero- and self-violence [13,33], such as drug use or suicidal ideation and gestures [34,35,36,37,38,39]. 

Therefore, sexual violence within prisons is a serious public health problem that requires targeted interventions to prevent and alleviate its health and social consequences worldwide [34]. In this regard, adequate forensic evaluation is a key element in determining sexual abuse in custody and identifying further preventive measures.

### 3.3. Psychological Abuse and Mental Health

Psychological abuse involves humiliation, threats, and degrading treatment. Sensory deprivation, overexposure, and sexual advances are other nonphysical methods. Despite the debate regarding the legitimacy and ethics of psychological interrogation methods, as mentioned by Punamaki et al., little research is available on their specific mental health consequences [40]. 

According to the Istanbul Protocol investigation and documentation of torture, common psychological reactions to torture may include reliving the trauma; emotional avoidance of any thought, conversation, activity, place, or person that elicits a memory of the trauma; profound personal detachment and social closure; forgetting an essential aspect of the trauma; hyperarousal (such as insomnia, irritability, difficulty concentrating, hypervigilance, an exaggerated startled response, generalized anxiety, shortness of breath, sweating, dry mouth or dizziness, and gastrointestinal disturbances); symptoms of depression; anhedonia (a marked decrease in interest or pleasure in activities); impaired appetite or weight loss; insomnia or hypersomnia; psychomotor agitation or retardation; fatigue and loss of energy; feelings of worthlessness and excessive guilt; difficulty in paying attention, concentrating, or remembering; thoughts of death and dying; suicidal ideation or suicide attempts; impairment of self-concept and loss of future perspective; dissociation, depersonalization, and atypical behavior; somatic disturbances, such as pain, headaches, or other physical complaints; sexual dysfunction, which is typical among torture survivors, particularly those who have experienced sexual torture or rape; psychosis with delusions, auditory, visual, or tactile disturbances; bizarre ideation and behavior, illusions or perceptual distortions, or paranoia and persecution mania; and substance abuse, such as alcohol and drug abuse [12,41]. 

Ehlers et al. conducted a study on former political detainees including torture survivors, asylum seekers, and refugees in politically safer societies across different countries (Europe, Asia, and the Middle East), showing increased levels of post-traumatic stress disorder (PTSD), depression, anxiety, and somatic and chronic pain disorders with varying rates of prevalence [42]. This study agreed with other data in the literature on prisoners [43,44,45,46,47,48]. For example, Tol et al. found a 60% rate of prevalence of PTSD and an 80% rate of prevalence of clinical depression and anxiety among torture survivors in Nepal [44]. Lower levels of PTSD have been observed in epidemiological or primary healthcare settings. For example, the prevalence rate was 14% among torture survivors in Bhutan and 20% among Latino refugees in the United States [44]. The link between the nature and severity of experiences of torture and ill treatment, as well as PTSD symptoms, was studied in 550 male non-help-seeking Palestinian political ex-prisoners from the Gaza Strip [45]. The results showed that the intensity of intrusive re-experience, withdrawal and numbness, and hyperarousal was directly related to the amount of exposure to physical, chemical, and electric torture; psychological ill treatment; and sensory deprivation or bombardment. However, the study findings showed that existential problems were unrelated to torture experiences. Furthermore, the duration of imprisonment; health problems during detention; harassment during arrest and after release; and family, marriage, and economic difficulties were predictive factors for intrusive traumare-experiences. In addition, ex-prisoners who continued to be ill-treated by military authorities and had financial problems were more affected by withdrawal, numbness, and hyperarousal than others [45].

PTSD is a diagnosable syndrome that an evaluating physician or psychologist can treat biologically and psychologically by attempting to relate to the individual’s mental suffering in the context of the individual’s beliefs and cultural norms. This includes respect for the individual’s political context and cultural and religious beliefs [12]. 

Physical and psychological forms of torture, especially when combined, are dangerous to prisoners’ mental health, as evidenced by the high rate of PTSD symptoms, and the literature data confirm that survivors of prison violence may also be at an increased risk of developing PTSD [49,50,51,52,53]. 

PTSD is the primary trauma-related diagnosis in The Diagnostic and Statistical Manual of Mental Disorders (4th ed., text revision, DSM-IV-TR; American Psychiatric Association) [54]. The diagnostic criteria for PTSD include having experienced, witnessed, or coped with one or more events that involved a threat of death or severe injury or a threat to the physical integrity of oneself or others and a response involving intense fear, helplessness, or horror, as well as the re-enactment of the traumatic event, avoidance of memories of the trauma, and numbing of general responsiveness. Boeschen et al. highlighted that more rape victims meet the diagnostic criteria for PTSD than victims of any other type of torture [55]. In particular, Kilpatrick et al. stated that repeated sexual assaults in prison are associated with an increased risk of PTSD [56]. 

In a report, Young suggested that among prisons, psychological violence related to male rape is exacerbated by the widespread belief that a “real man” cannot be forced into sexual violence against his will. Therefore, the victim must have necessarily given consent to the assault [57]. A recent work highlighted that the society is still silent on the issue of male–male rape, mainly because sexual activity between two men is often interpreted as indicative of homosexuality [58]. In the prison context, this cycle can be amplified because the man who has been raped is perceived as symbolically emasculated and is at risk of further victimization. For many male victims, the perceived loss of manhood and the resulting humiliation are psychologically destructive. As mentioned above, the mental health correlates of prison rape victims are not well-understood and lack diagnostic specificity. Therefore, applying the treatments used for female rape victims to male victims of prison rape, without modification, could be misleading. Therefore, much work remains to be done in order to identify empirically appropriate treatments for prison rape victims.

The diagnosis of PTSD among male rape survivors does not include all the typical post-rape symptoms identified in female victims, such as depression, anger, sexual dysfunction, guilt, humiliation, and alterations incore belief systems about the self and others, which are familiar to many victims [55,59]. Most research on the treatment of rape-related mental trauma has been conducted on female victims of sexual assault. In this regard, Kilpatrick et al. [56] recommended that secondary prevention strategies (e.g., psychosocial and pharmacological treatments) should be implemented within a short time after the trauma (e.g., within four weeks) to mitigate the occurrence of trauma-related psychological or psychiatric symptoms. As outlined by Kilpatrick et al. [56], specific treatments of these victims for PTSD include exposure therapy, cognitive therapy, anxiety management training, and psychoeducation. The literature on pharmacological interventions has shown that drugs such as propranolol and selective serotonin reuptake inhibitors (SSRIs) reduce symptoms related to the disorder to a lesser degree than cognitive-behavioral psychotherapy.

Evidence in the literature suggests that the treatment used in prison should be appropriate to the trauma suffered, individualized (e.g., including the victim’s specific experiences based on his/her gender and sexual orientation), culturally sensitive, of sufficient duration to treat the victim, adequate, evidence-based, and holistic, with members of the healthcare team working together for the victim [49,50,51,52]. Victims are also at an increased risk of depression and suicide, as those who experience repeated violence develop a sense of helplessness and fear to the extent that they consider suicide their only option [49,50,51,52]. Psychological torture has also been associated with somatic symptoms typical of survivors, such as weight loss, hypertension, and pain. One possible reason why psychological torture is related to bodily discomfort is that the conditions of captivity prevent any emotional expression (including the expression of feelings). According to Fields et al. [60] and Basolglu et al. [61], psychological violence may pose a risk of prolonged pain and somatic diseases through altered brain memory and executive functions. All this poses a high health risk for prisoners. Humiliation, psychological torture, and degrading treatment cause pain and suffering, thus meeting the criteria of torture, and should be explicitly prohibited under international law. Although scientific data show that physical and psychological torture harm prisoners’ mental health, the literature also shows that prisoners with mental illnesses are more likely to be abused.

Since many forms of maltreatment leave no physical outcomes or might be nonspecific, with little evidentiary value, it is undeniable that the absence of physical injuries or mistreatment does not evidence a lack of injustice. As traditionally described in the forensic literature, prisoners may often inflict severe injuries on themselves (self-injury) in various ways [3,4,5,6,62]. In the forensic field, it is essential to distinguish between torture and self-injury outcomes, particularly in prisoners with psychiatric diseases [3,4,5,6]. 

### 3.4. Victimization of Prisoners with Mental Illnesses

As per the scientific data, physical and psychological torture harms inmates’ mental health, and inmates with mental illnesses are more likely to be mistreated [13]. 

Blitz et al. showed that people with psychiatric disorders continue to be overrepresented in prisons and jails, despite an increase in the number of programs, such as specialized law enforcement responses, prison diversion programs, mental health courts, and different reintegration services, aiming to reduce their involvement in the correctional system [10]. 

Traumatic victimization, particularly sexual abuse, has been consistently identified as a serious problem in prisons worldwide. Individuals with psychiatric illnesses, such as schizophrenia and bipolar depression, being vulnerable, are up to eight times more likely to be victims of sexual abuse than prisoners without mental illness [10,34,63,64,65,66]. 

A 2009 report on the prevalence of severe mental illness in prisons showed rates of 14.5% among male inmates and 31% among female inmates at that time [67]. These rates increased to 17.1% in men and 34.3% in women when post-traumatic stress disorder (PTSD) was included. According to Silver et al. [68], there are two “theories” about why detainees with mental illness have a higher risk of victimization. The first hypothesis, enhanced vulnerability to attack, explains that this is because inmates are confused by drugs, with attenuated responses, and are therefore vulnerable and unable to protect and defend themselves [33,69]. The second hypothesis, victimization as an informal social control, attributes the increased risk to behaviors such as illogical thinking, delusions, auditory hallucinations, and severe mood swings among people with SMI that may be disquieting for other non-ill inmates or correctional staff and result in violent attempts, on their part, to control or reduce such behaviors [47]. Both hypotheses suggest that a symptomatic inmate may be susceptible to further violence. A significant correlation has been observed between symptom severity and subsequent physical and sexual abuse [70,71]. Blitz et al. [10] also found higher rates of inmate-on-inmate physical violence among male and female inmates with serious mental illnesses compared with non-ill inmates. Male inmates with mental disorders were 1.6 times more likely than their counterparts to be physically assaulted by another inmate. The rate of victimization of females with a mental disorder was 1.7 times higher than that of females with no mental disorder. Among men with mental illnesses, individuals with schizophrenia or bipolar disorder were at the highest risk (310 per 1000 prisoners).

Although research on the prevalence of victimization among people with mental illnesses in the community is extensive and shows extremely high rates of trauma (91%) and PTSD (19%) [72,73], empirical data on the prevalence of victimization among prisoners with mental illnesses are lacking.

Therefore, new research is needed to expand the empirical literature so as to improve our understanding of the problem and implement judicial, institutional, and public health surveillance worldwide [13]. 

### 3.5. Prison Torture and Autopsy Findings

In the case of a custodial death, the coroner must identify the causal relationship between torture and death through an autopsy. In the literature, there are no systematic studies but only isolated case reports describing autopsy findings in torture cases [16,74,75,76,77,78]. Fineschi et al. first described the “incaprettamento”, a homicide method used by the Italian Mafia, as having admonitory significance and being frequently related to torture signs on the victim’s body [74,75]. According to Pollanen, forensic pathology can be used in autopsy to identify injuries of different severities [76,77]. On the basis of the cases observed during international missions, Pollanen identified the three most frequent methods of inflicting trauma during torture: the use of blunt force, use of electrical and thermal currents, and method of inflicting injuries by suspension or stress positions. In this regard, suspending the body by the upper or lower limbs (a variant of “incaprettamento”) can result in prolonged, forced, unnatural positions, leading to a fatal outcome. Meel [78] discussed the history, physical examination, treatment, and autopsy findings of fatal pulmonary thromboembolism following physical torture. He concluded his work by suggesting that doctors caring for torture victims be alerted to the occurrence of renal failure and myoglobinuria, and that forensic pathologists be alerted to the possible complications of torture by venous thromboembolism. The primary recommendations in this field include the revised Minnesota Protocol of the United Nations [78,79] and the Autopsy Protocol of the International Committee of the Red Cross [80]. Furthermore, the Istanbul Protocol [12,81] is an essential guide for approaching the postmortem examination of cases of torture and maltreatment in custodial settings. A complete traditional medical autopsy consists of a comprehensive and standardized dissection of the body. Concerning the autopsy technique to be used in cases of torture, maltreatment, and death in custody, on the basis of the injury distribution, additional dissections, such as musculocutaneous and nerve dissection of a specific anatomic region involved in the trauma, may be helpful [82]. 

Layered dissection of the head can show serious evidence of trauma, such as facial fractures. In addition, layered neck dissection may be necessary to detect specific injuries caused by asphyxiation and compression of the neck. Head–neck dissection should be performed after the evisceration of the thoracoabdominal organs and removal of the encephalon to enable blood drainage from the large veins of the thoracic and cranial districts. This procedure decreases the risk of iatrogenic bleeding during dissection. In suspected cases of falanga, foot dissection is recommended [82]. 

An often-unrecognized trauma pattern arising in fatal torture cases involves the musculoskeletal system. Such injuries may escape detection, given that the major joints are not usually examined during autopsy. In these cases, the major joints must be dissected to garner essential information about the nature of trauma sustained by the upper arms or the lower limbs. The specific signs of torture and mistreatment of detainees most frequently detected by autopsies are due to blunt force injuries, as observed in prisoners who have survived torture. The injury most frequently detected during autopsy is tram track hematoma due to impact with an elongated rigid or semi-rigid implement. The scars are characterized by patterned areas of discoloration on the skin and must be differentiated from other dermatological diseases. Patterned weapons can cause superficial lacerations and infection, which can delay the healing process [81,82,83,84,85]. 

As mentioned before, periodic blunt force injuries to the feet can cause subcutaneous hemorrhages in the feet [83]. Falanga, as this method of torture is called, may escape detection during external inspection. It is important not to confuse the minor bruises caused by the falanga method with postmortem hypostasis [81,82,83,84,85]. 

A less frequently documented method of torture and maltreatment among prisoners is the use of suspension and stress positions. A simple stress position is complete suspension from the wrists using cuffs or ligatures. At autopsy, ligature marks are visible on the areas involved, such as the wrists. Ligature may lead to ischemic necrosis of the hands, including the development of frank gangrene. Ligature marks may provide information that help the investigator to assess the duration of the suspension. Signs of thermal injuries, such as thermal burns (mostly second-degree), can be observed during the autopsy and are variably well-demarcated. They are often circular and localized to the digital pads of the toes and fingers or the back of the hand. However, they can be observed in any anatomical district. Electrical injuries can be observed during autopsy. However, it can be challenging to observe electric marks due to the presence of clothes [81,82,83,84,85]. 

Regarding the cause and manner of deaths associated with torture in prison, four main “patterns” of death can be identified. In the case of acute death, torture-related injuries can be immediately fatal. Typically, malignant pathophysiology is related to hemorrhagic shock, embolism, sepsis, or renal failure. Death can be secondary to neglect in the management of the prisoner as a consequence of poor conditions of detention, negligence, or airborne infections (or infections due to poor custodial hygiene) or a lack of healthcare for an acute or chronic illness. The prisoner may be subjected to food and water deprivation. Malnutrition and starvation may lead to a fatal infection (bronchopneumonia), or dehydration with subsequent rehydration may have neurological consequences. Finally, even if the detainee was tortured, his/her death may be unrelated to the mistreatment, as in cases of death by suicide [79,80,81,82,83]. 

Autopsies must be performed on all prisoners who die in custody to establish the role of torture in the victim’s death. In recent years, humanitarian forensic science has emerged as a growing forensic discipline. This platform applies the various forensic disciplines to determine the truth of the injury, death, and disappearance of people during times of war, internal armed conflict, and terrorist actions. This knowledge, in turn, helps to improve humanitarian actions so as to prevent recurrence. Autopsies conducted in cases of torture, death in custody, and extra-judicial execution have expanded the evidence base regarding the nature of injuries and causes of death among people killed in those circumstances. Allegations of maltreatment in custody necessitate one to investigate. In singular cases, maltreatment can be proven. Sometimes, it can be deemed probable or improbable, and rarely, maltreatment can be excluded. The investigation has to include a determination of whether the maltreatment could have been life-threatening or possibly lethal, and autopsy could be invalid. To harmonize the implementation of autopsy in this field, standardized protocols should be encouraged, given the importance of autopsy in providing proof of maltreatment in custody.

## 4. Discussion

The selected studies show that maltreatment in prison is a relevant and debated forensic issue. Maltreatment in prison can coincide with torture [18]. Torture is described as one of the most extreme forms of violence [83]. The lifetime prevalence rates of physical and sexual violence are higher among inmates, at 6 months and 12 months of incarceration, than among non-prisoners. In addition, women report much higher rates of traumatic events than men [13]. Torture, maltreatment, and extrajudicial executions of prisoners have occurred throughout history. However, the medical and forensic investigation of torture has been described only recently [3,18,79,80,81,83]. 

Methods of ill treatment are traditionally divided into the physical and psychological, but physical processes inevitably have psychological consequences. Some ill treatment methods, such as sexual violence, almost unavoidably combine the physical with the psychological [3]. Physical and psychological torture methods, especially when combined, are dangerous for prisoners’ mental health, as evidenced by data in the scientific literature [47,48,49,50]. The ill treatment and torture of detainees constitute a global problem, and there is significant geographic variability in the physical methods used. Several geographic differences in the methods used were observed in epidemiological studies regarding torture in prison. Some, such as falanga (beating on the soles of the feet), are relatively specific to well-defined regions of the world, e.g., the Middle East [15]. Some are country-specific; for example, interrogation by shaking is used in Israel [16], and plastic bag asphyxiation is used by the Spanish anti-terrorist police [17]. Maltreatment in custody depends on national legislation, international conventions, control by international organizations and local activists, and reporting by international and national media influence. Sometimes, a prisoner’s death impedes the provision of testimony by the prisoner or performance of a forensic investigation. In some countries, the scientific organization can be restricted in the assessment of physical and psychological violence in prisons by the provisions of local governments [84,85,86], thus limiting the scientific contributions existing in the literature and the positive impact of forensic investigation in the development of further prevention measures. Knowledge regarding which torture methods are practiced in any given part of the world can be greatly beneficial when examining a possible torture victim since, while some forms of torture leave obvious signs, others can be difficult to document. The Istanbul Protocol specifically states in Chapter IV, Section A, Article 122, that any medical examiner of torture should be prepared to both correlate the degree of consistency between examination findings and specific allegations of abuse by the patient and correlate the degree of consistency between individual examination findings with the knowledge of torture methods used in a particular region and their common aftereffects [4,5,6]. In many cases, torturers deliberately try to avoid leaving any visible traces of the torture they inflict. It is not unreasonable to assume that having an understanding of the types of torture prevalent in a certain part of the world can minimize the risk of overlooking or misinterpreting signs of abuse. Therefore, knowledge of the geographical patterns of different forms of torture is a valuable resource for the examiner, given that it is necessary to establish a relationship of trust, understanding, and empathy between the examiner and victim so as to minimize the risk of underreporting. Moreover, awareness of the geographical patterns of torture in prisons may be helpful in determining further local public health and policy prevention measures. 

In the medical literature, most of the published contributions on torture are based on retrospective studies of torture among survivors. These survivors are often asylum seekers [15,81,85,86]. The humane treatment of a detainee with respect cannot be dependent on the material resources available to the state party [1]. All countries must adequately secure all detainees’ physical and psychological integrity and well-being. This responsibility includes a duty of care and the adoption of preventive measures aiming to protect the most vulnerable detainees and to reduce the risk of violence inflicted by other inmates [1]. In some custodial settings, there is a trend toward the outsourcing of logistics, services, and operations to private companies. Outsourcing can concern various areas, from catering, prison shops, and work to detainee transfers and custody services. In some contexts, prisons may be entirely run by private companies. Whatever the sector and degree of privatization of prisons, the state remains fully accountable if there is any violation of the prohibition of torture and ill treatment [1]. In addition, information regarding the autopsy findings of torture victims is still lacking, which maybe a consequence of a low rate of autopsies in the case of torture, partly because torturers often dispose of the victims’ bodies. Otherwise, when autopsies are performed, the examinations are frequently not completed due to limitations on the documentation of physical findings. Indeed, the scientific literature on autopsy findings of torture is often limited to case reports in this field [16,74,75,76,77,78]. As a result, the cause of death of a prisoner is not always clear, which contributes to impunity and a lack of awareness about the detailed medical and forensic aspects of torture [81]. Since many forms of maltreatment have no physical outcomes or may be non-specific, with little evidentiary value, the absence of physical injuries of mistreatment does not necessarily indicate a lack of mistreatment. As traditionally described in the forensic literature, prisoners may often inflict severe injuries on themselves (self-injury) in various ways [3,62]. This frequently occurs because the incidence psychiatric disorders is higher among prisons than in the general population [3,87]. Moreover, psychological maltreatment may not be related to physical signs of abuse. In the forensic field, it is essential to distinguish between torture and self-injury outcomes [3]. Seeking the truth regarding the medical consequences of torture will increase our knowledge about torture-related injuries, contribute to the rehabilitation of torture survivors, and strengthen humanitarian forensic action, given that humanitarian forensics has emerged as a growing forensic discipline in recent years [81]. When dealing with torture in custodial settings, a multidisciplinary approach is required, involving mutual cross-talk between all the forensic disciplines to improve the quality of maltreatment investigations and effectively implement further interventions [88,89,90,91,92]. 

In order to improve the protection of the human rights of inmates, a prison system should be managed in a fair and humane manner, and national legislation, policies, and practices must be guided by the international standards. Prison authorities have a responsibility to ensure that the supervision and treatment of prisoners are in line with the rule of law with respect to individuals’ human rights and that the period of imprisonment is used to prepare individuals for life outside prison following release. Nevertheless, national legislation and rules relating to the management of prisons are outdated and in need of reform. In many countries, prison departments are under the authority of police or military institutions, and managers and staff receive no specific training regarding prison management. Staff morale is usually low, and effective leadership, serving to drive prison reform, is lacking. One strategy which seeks to prevent ill treatment and encourages the adoption and respect of legal norms and standards that prohibit torture and combat impunity is to monitor places of detention through periodic inspections and monitor how human rights obligations are met. Such visits, carried out by internal, juridical, and independent organizations such as official institutions established by parliaments, bodies attached to a specific ministry or civil society groups, or a mixture of these, offer an effective way to prevent violations of human rights among persons deprived of their liberty, including the most egregious violations in the forms of torture and inhuman or degrading treatment, and improve the conditions of detention [93]. 

## 5. Conclusions

This review presents and discusses data regarding the forensic issues of maltreatment and torture in custodial settings. The maltreatment and torture of prisoners constitute a severe problem worldwide. In the medical literature, most of the published data on torture are based on retrospective studies of torture among survivors and often refer to asylum seekers. It is common to observe chronic physical outcomes or psychological complications. For this reason, acute physical injuries are rarely observed, and detailed data on torture implemented in prisons are lacking. In addition, in some countries, regulations developed by local governments can restrict scientific organizations that aim to assess physical and psychological violence. Consequently, these governments limit the breadth of scientific contributions existing in the literature. Forensic evaluation is crucial for assessing the elements evidencing torture and maltreatment, both physical and psychological. A multidisciplinary approach and standardized, updated methodologies are needed to support policymakers, national institutions, and public health system initiatives in this field. Further research is required to estimate this phenomenon in depth and prevent it, especially in low-income countries. To improve the human rights of inmates who face maltreatment and torture in prisons, it is crucial to improve prison regulations designed to document the health status of prisons, together with the full integration of the national health systems in penitentiary institutions, increased surveillance conducted by supranational institutions, and the implementation of training for prison staff. Therefore, in forensics, the medico-legal evaluation of prisoners is currently an open challenge.

## Figures and Tables

**Table 1:** Most frequent methods of maltreatment and torture.

Maltreatment Method	Common Findings
Blunt impact trauma	Physical injuries are often nonspecific, but hits with batons, electric cables, and sticks commonly leave classic “track” bruises that can leave permanent hypo- or hyperpigmented scars.
Falanga	Superficial hemorrhage occurs in the soles of the feet, with a possible extension in the dorsum of the feet. Large bruises on the feet, with deformity, are rare.
Electric injury	Electric marks are reddish-brown in color, with inflamed margins, before they darken, resolve completely, or leave thin white scars, which are punctiform when a wire end has been used. These might be partially circumferential, wrapping around a finger, if the wire was wrapped around it. Clusters of punctiform scars (picana) in unusual locations, including the toes or the foreskin, are strongly suggestive and typical of maltreatment using electric current. They are small and, therefore, they may remain undetected unless explicitly pointed out by the detainee.
Asphyxiation	It usually leaves no marks, and recovery is rapid. Only in rare cases can it result in fractures of the larynx or softtissue scarring.
Stress position	All forms of suspension or ligature involving limb ligation can cause scars and ligature marks of specific types and in specific locations.Musculoskeletal and nerve injuries can also occur.
Thermal injuries	Cigarette burns often leave 5 to 10 mm-long circular or ovoid macular scars. They may have a hyper- or hypopigmented center and a hyperpigmented, relatively indistinct periphery. Burning with hot objects produces markedly atrophic scars that reflect the shape of the tool and are sharply demarcated by hypertrophic or hyperpigmented marginal areas that relate to the initial zone of inflammation. The burn may result in hypertrophic or keloid scars, as in the case of a burn caused by burning rubber.

## Data Availability

Data sharing is not applicable; no new data were created or analyzed.
